# Phenotypic and Genetic Characterization of a Cohort of Pediatric Wilson Disease Patients

**DOI:** 10.1186/1471-2431-11-56

**Published:** 2011-06-17

**Authors:** Tawhida Y Abdel Ghaffar, Solaf M Elsayed, Suzan Elnaghy, Ahmed Shadeed, Ezzat S Elsobky, Hartmut Schmidt

**Affiliations:** 1 Yassin Abdel Ghaffar Charity Center for Liver Disease and Research, Cairo, Egypt; 2 Children's Hospital, Ain Shams University, Cairo, Egypt; 3 Medical Genetics Center, Cairo, Egypt; 4 Klinische und Experimentelle Transplantationshepatologie, Universitätsklinikum, Münster, Germany

**Keywords:** hepatic, mutations, neurological, pediatric, Wilson disease

## Abstract

**Background:**

In Egypt, Wilson disease seems to be under diagnosed and clinical data on large cohorts are limited. The aim of this study is to highlight the clinical, laboratory and genetic characteristics of this disease in our pediatric population as well as to report our experience with both treatment options and outcome.

**Methods:**

The study included 77 patients from 50 unrelated families (62 were followed up for a mean period of 58.9 ± 6.4 months and 27 were asymptomatic siblings). Data were collected retrospectively by record analysis and patient interviews. Diagnosis was confirmed by sequencing of the *ATP7B *gene in 64 patients

**Results:**

Our patients had unique characteristics compared to other populations. They had a younger age of onset (median: 10 years), higher prevalence of Kayser-Fleischer rings (97.6% in the symptomatic patients), low ceruloplasmin (93.5%), high rate of parental consanguinity (78.9%) as well as a more severe course. 71.42% of those on long term D-penicillamine improved or were stable during the follow up with severe side effects occurring in only 11.5%. Preemptive treatment with zinc monotherapy was an effective non-toxic alternative to D-penicillamine. Homozygous mutations were found in 85.7%, yet limited by the large number of mutations detected, it was difficult to find genotype-phenotype correlations. Missense mutations were the most common while protein-truncating mutations resulted in a more severe course with higher incidence of acute liver failure and neurological symptoms.

**Conclusions:**

Egyptian children with Wilson disease present with early Kayser-Fleischer rings and early onset of liver and neurological disease. The mutational spectrum identified differs from that observed in other countries. The high rate of homozygous mutations (reflecting the high rate of consanguinity) may potentially offer further insights on genotype-phenotype correlation

## Background

Wilson's disease (WD) is a rare autosomal recessive disorder of copper metabolism, with a prevalence of about 1 in 30 000 people. Its frequency increases in populations where consanguinity is more common [1]. It is characterized by decreased biliary copper excretion and defective incorporation of copper into ceruloplasmin, leading to copper accumulation in different tissues mainly the liver, brain and kidneys [2].

WD typically begins with a pre-symptomatic period, during which copper accumulation in the liver causes subclinical hepatitis and progresses to liver cirrhosis and development of neuropsychiatric symptoms. The type of hepatic and neurological symptoms can be highly variable. It may also present as fulminant hepatic failure with an associated Coomb's-negative hemolytic anemia and acute renal failure [3]. If untreated WD causes progressive fatal liver and brain damage [4] and therefore, early recognition and treatment is required.

In 1993, the WD gene, *ATP7B, *was cloned. This gene codes for a membrane-bound copper -transporting ATPase expressed primarily in the liver [5,6]. More than 400 mutations within the *ATP7B *have been identified so far (personal data). Although molecular genetic diagnostics are increasingly available for clinical use, in practice their use is very limited.

The diagnosis of WD is based on the results of several clinical and biochemical tests, each has its limitations, and only the combination of clinical, biochemical and genetic tests provides a powerful and reliable tool for the diagnosis [3].

Zinc sulfate and D-penicillamine (D-PCA) have both proved to be clinically effective in the treatment of WD, but zinc sulfate offers the advantage of having a very low toxicity, especially in the case of neurological involvement. For asymptomatic or pre-symptomatic patients, zinc salts or maintenance dosages of chelating agents may be used [7].

In Egypt, where viral hepatitis (HCV, HBV) is by far the major player in the field of liver disease, WD seems to be under diagnosed and clinical data on large cohorts are limited owing to its low frequency, being a rare disease. The aim of this study was to highlight the clinical, laboratory and genetic characteristics of WD in our pediatric population as well as to report our experience with both treatment options and outcome aiming at increasing awareness about diagnosis and management. Based on the mutational spectrum rapid screening approaches may be applied for Egypt in the future.

## Methods

The study included 77 WD patients (75 children and 2 adult cousins) (43 males and 34 females), 62 of whom (34 males and 28 females) were followed up for a mean period of 58.9 ± 6.4 months. Patients were from 50 unrelated families presenting to Yassin Abdelghaffar Charity Center for liver disease and research and the Pediatric Hepatology clinic, Ain Shams University (both are tertiary referral centers) from 1992 to 2009. One or more authors cared for all patients. Data were collected retrospectively by record analysis and patient interviews.

The study was approved by the ethical committee of both Yassin Abdelghaffar Charity Center for liver disease and research and Ain Shams University and informed consent for inclusion in the study was obtained from parents of children.

Initial diagnosis of WD was made if the patient had hepatic and/or neurologic disease in addition to at least two of the following six criteria (adapted and modified from Dhawan et al., 2005)[8]:

1- Positive family history of WD

2- Low ceruloplasmin level (< 20 mg%)

3- Presence of Kayser-Fleischer (KF) ring

4- Liver biopsy suggestive of WD (positive staining of copper associated protein (rhodanine or orcein stain), presence of glycogenated nuclei, micro- or macrovesicular steatosis, or ultra structural changes defined by electron microscopy) as measurement of liver copper is not available in Egypt.

5- Elevated baseline 24-hour urinary copper excretion (more than 100 μg/24 hours or more than1600 μg/24 hours after D-PCA challenge test).

6- Coomb's negative hemolytic anemia

According to the type of presentation, symptomatic patients were classified into three groups:

- **Group 1 (G1)**: Patients with *hepatic presentation *defined as presence at time of diagnosis of exclusively hepatic symptoms and signs in the absence of neurological symptoms and signs as confirmed by careful neurological examination (n = 35).

- **Group 2 (G2)**: Patients with *neurological presentation *defined as the presence of neurological symptoms and the absence of hepatic symptoms at the time of diagnosis (n = 6).

- **Group 3 (G3)**: Patients with *combined presentation *defined as the appearance of neurological manifestations in a patient with hepatic disease within the first 6 months of the initial diagnosis (n = 9).

Brothers and sisters of all patients diagnosed with WD were screened for the disease by being subjected to full history taking, physical examination, serum ceruloplasmin, liver function tests, slit lamp examination for KF ring, and molecular testing. Those who had no symptoms at presentation and were discovered to have WD during screening of family members of the index case comprised **Group 4 (G4)**: *Asymptomatic cases *(n = 27).

Patients with liver symptoms (both G1 and G 3) were then re-classified according to type of hepatic presentation into:

- *Acute hepatitis (AH): *defined as acute onset of jaundice and elevated liver enzymes in a previously apparently healthy subject (n = 6).

- *Acute liver failure (ALF)*: defined as coagulopathy (INR > 2) with acute onset of jaundice with or without encephalopathy (n = 13).

- *Chronic liver disease (CLD): *defined as patients with any form of chronic liver disease (compensated or decompensated) (n = 20).

Liver function tests and other routine laboratory data (including viral markers and autoantibodies) were performed using standard methods to evaluate the degree of liver affection as well as to identify/exclude any associated hepatic condition. If not contraindicated due to severe coagulopathy or advanced refractory ascites, liver biopsy was obtained (n = 39) before initiation of treatment. MRI brain was performed in ten patients with neurological symptoms

Diagnosis was confirmed by mutational analysis (sequencing of *ATP7B *gene) in 64 patients (25 asymptomatic and 39 symptomatic) (48 of whom were previously reported in Abdelghaffar et al., 2008 study) [9].

### Treatment

• Group 1 patients:

- In all patients D-PCA was initiated in incremental doses, starting with 2-5 mg/kg/day and gradually increasing the dose every week (after testing the urine for RBCs and albumin and doing a CBC) to a maximum of ~20 mg/kg/day in two divided doses (mean dose was 18 mg/kg/day).

- Zinc sulfate was given (as zinc acetate is not available in Egypt) in the initial period in a dose of 75 -150 mg/day in two divided doses till the optimal dose of D-PCA was reached and then gradually decreased and maintained at a single daily dose of 50-75 mg. Patients were instructed to leave at least 6 hours between the doses of zinc and D-PCA.

Maintenance therapy was started in patients after becoming clinically well with stable or normal transaminases and hepatic synthetic function and 24-hour urinary copper less than 500 μg/day. D-PCA dose was gradually decreased and then maintained at a dose of ~10 mg/kg/day and zinc dose was maintained at 50- 75 mg/day.

• Group 2 and Group 3 patients:

D-PCA was started as in group 1 but maximum dose reached was lower (maximum 7 mg/kg/day) and zinc sulfate was given at 150-225 mg/day.

• Group 4 patients:

The regimen of group 1 was applied to all asymptomatic children except three patients who had only elevated liver enzymes and for whom zinc sulfate monotherapy (given in three daily doses) was used. One of them was only one year old.

D-PCA was discontinued when serious side effects occurred, in which case zinc sulfate was the only treatment used. Vitamin E in a dose of 200-400 IU was prescribed to all patients while vitamin B6 was prescribed to all patients taking D-PCA. All patients were advised to follow a low-copper diet.

### Follow up and compliance

Patients were seen regularly every 7-10 days until the optimal dose of D-PCA was reached, thereafter every month for three months then three to four times per year. In each visit, they were assessed clinically and their ALT, albumin, INR and CBC were determined, urine examination was done and drug adverse effects were checked for. Doppler abdominal U/S and examination for KF ring were performed every year or as needed.

Compliance was assessed by interviewing the patients and their parents, regular checking of transaminases and by yearly assessment of free serum copper and 24 hour copper in urine. Disappearance of a previous KF ring and normalization of free serum copper were taken as evidence of compliance. Non adherence (non compliance) was defined as not taking medication as prescribed associated with increased liver transaminases, urinary copper or free serum copper (modified after Arnon et al., 2007) [10].

## Statistical methods

The standard computer program SPSS for Windows, release 13.0 (SPSS Inc, USA) was used for data entry and analysis. All numeric variables were expressed as mean ± standard deviation (SD). Comparison of different variables in various groups was done using student's t test and Mann Whitney test for normal and nonparametric variables, respectively. Multiple regression analysis was also performed to determine effect of various factors on a dependent variable. Chi-square (χ^2^) test was used to compare frequency of qualitative variables among the different groups. Spearman's correlation test was used for correlating non-parametric variables. For all tests a probability (p), less than 0.05 was considered significant. Survival statistics was done using the Kaplan Meire curve [11].

## Results

### Patients' features at disease presentation

Mean age of the 75 pediatric patients at disease onset was 9.92 ± 0.37 years (Median: 10 years, range 1-18 years) (excluding the two adult patients). Parental consanguinity was present in 78.9% of families (table 1).

**Table 1:** Demographic data and type of presentation of the studied group

*Age of onset (years)*	*Range*	1-18
	*Mean ± SD*	9.92 ± 0.37
	*Median ± IR*	10 ± 4
***Age at presentation (years)***	***Range***	1.0 -19
	***Mean ± SD***	10.96 ± 0.45
	***Median ± IR***	10 ± 4

*** Sex (M/F)***		43/34

*** Consanguinity No. (%)***		56/71 (78.9%)

*** Number of families***		50

***Residency (73)- No. (%)***	*** Upper Egypt***	25 (34.2%)
	***Lower Egypt***	30 (41.1%)
	***Cairo***	12 (16.4%)
	***Non-Egyptian***	6 (8.2%)

***Type of presentation No. (%)***	*** Hepatic***	35 (45.5%)
	***Neurological***	6 (7.8%)
	***Combined***	9 (11.7%)
	***Asymptomatic***	27 (35.1%)

N.B. Two adult patients with ages of 30 and 37 years (cousins of one of the children) were excluded when calculating the age ranges and mean.

M: male, F: female, SD: standard deviation, IR: interquartial range

Leipzig score [12] was retrospectively applied to the patients: 73 of them had a score of ≥4 and only 4 children had a score of less than 4 (two asymptomatic and two hepatic); none of whom did mutational analysis at the time of scoring.

Serum ceruloplasmin was <20 mg/dl in 93.5%. Elevated urinary copper excretion was present in 19/25 (84.2%), KF ring was detected in 45/65 (69.2%) and positive family history of WD was present in 77.8% of patients (table 2).

**Table 2:** Criteria used in diagnosis of WD in the studied group

		**No**.	%
***S. Ceruloplasmin (mg%)***	**≤ *7***	** 57/77**	74
	***7-20***	**15/77**	19.5
	***> 20***	**5/77**	6.5

***Positive Family history***		**56/72**	77.8

***K F ring***		**45/65**	69.2

***Elevated urinary copper excretion***		**19/25**	84.2

***Liver biopsy suggestive of WD***		**15/39**	38.5

***Coomb's negative hemolytic anemia***		**4/74**	5.4

The mean age at presentation was 10.96 ± 0.45 years (Median: 10 years). Patients with hepatic manifestations (G1) showed earlier onset than those with neurological manifestations (G2 and G3), (median age of onset = 10, 12, 11 years respectively), a difference which was statistically significant (*p *= 0.017). Family screening was effective at diagnosing WD slightly earlier at a median age of 8 years (table 3)

**Table 3:** Comparison between different studied groups as regards clinical and laboratory data

		G1 (35)	G2 (6)	G3 (9)	G4 (27)	Total (77)	P value
***Male/Female***		20/15	4/2	7/2	12/15	43/34	0.32

***Median age of onset (yrs) (Range)***		10 (6-15)	12 (10-14)	11 (9-18)	8 (1-17)	10 (1-18)	0.017 *

***Duration between onset and diagnosis (yrs)***		0.83 ± 3	4.75 ± 0.9	1.9 ± 0.6	-	1.03 ± 0.2	0.000*

***Duration of follow up (ms) Mean (Range)***		60.27 (0.25-156)	32.5 (12-48)	75.86 (12-156)	89.55 (5-156)	58.9 (0.25-156)	

***S. Ceruloplasmin***	***<7***	25 (71.4)	6 (100)	7 (77.8)	19 (70.4)	57 (74)	
***(mg%)***	***7-20***	6 (17.1)	0 (0)	2 (22.2)	7 (25.9)	15 (19.5)	
***No. (%)***	***>20***	4 (11.4)	0 (0)	0 (0)	1 (3.7)	5 (6.5)	

	***Hepatomegaly***	22 (62.9)	2 (33.3)	5 (55.6)	22 (81.5)	51 (66.2)	0.134
**Hepatic**	***Splenomegaly***	22 (62.9)	1 (16.6)	6 (66.6)	9 (33.3)	38 (49.4)	0.006
**signs**	***Jaundice***	24 (68.6)	0 (0)	2 (22.2)	0 (0)	26 (33.8)	0.000*
**No. (%)**	***Ascites***	17 (48.6)	0 (0)	4 (44.4)	0 (0)	21 (27.3)	0.000*
	***LL Oedema***	15(42.9)	0 (0)	5(55.6)	0 (0)	20 (26)	0.000*

	***Encephalopathy***	2 (5.7)	0 (0)	0 (0)	0 (0)	2 (2.6)	
	***ALT (x ULN*)**	2.8 ± 0.4	1.7 ± 0.8	1.7 ± 0.3	3.2 ± 0.5	2.7 ± 0.3	0.056
**Liver**	***AST (x ULN)***	3.9 ± 0.5	1.0	3.9 ± 0.2	2.4 ± 0.3	3.2 ± 0.3	0.001*
**function**	***Bilirubin (mg%)***	8.0 ± 1.7	2.5 ± 1.6	1.8 ± 0.5	0.8 ± 0.1	4.2 ± 0.8	0.000*
**tests Mean ± SD**	***INR***	3.0 ± 0.4	1.3 ± 0.2	3.8 ± 0.2	1.1 ± 0.04	2.3 ± 3.4	0.000*
	***Albumin (gm%)***	2.6 ± 0.1	4.3 ± 0.1	3.0 ± 0.3	3.9 ± 0.2	3.2 ± 1.2	0.000*

	***Chronic hepatitis***	4/11 (36.4)	1/4 (25)	0/4 (0)	7/20 (35)	12/39 (30.8)	
**Liver**	***Cirrhosis***	7/11 (63.6)	1/4 (25)	4/4 (100)	9/20 (45)	21/39 (53.8)	
**biopsy**	***Steatosis***	3/11 (27.3)	1/4 (25)	2/4 (50)	2/20 (10)	8/39 (20.5)	
**No. (%)**	***Glycogenated nuclei***	2/11 (18.2)	1/4 (25)	0/4 (0)	7 (35)	10/39 (25.6)	
	***Cu associated protein***	4/11 (36.4)	0/4 (0)	1/4 (25)	5 (25)	10/39 (25.6)	
	
	***Pigment***	1/11 (9)	1/4 (25)	0/4 (0)	2 (10)	4/39 (10.3)	

**Positive KF rings No. (%)**		27/28 (96.4)	6/6 (100)	8/8 (100)	4/23 (17.4)	45/65 (69.2)	0.000

Time lag between onset of the disease and its diagnosis was 1.03 ± 0.2 years; it was prolonged in patients with neurological symptoms (4.75 ± 0.9 years in G2 and 1.9 ± 0.6 years in G3) compared to the hepatic group (0.83 ± 3 years), a difference which showed a high statistical significance (*p *= 0.000).

Hepatomegaly was the most common sign present in asymptomatic patients (being found in 81.5%). It was present in 62.9% and 55.6% of patients in the hepatic and combined groups, respectively, but in only 33.3% of purely neurological cases. Splenomegaly was most common in G1 (62.9%) and G3 (66.6%). Surprisingly, one third of asymptomatic patients (33.3%) had splenomegaly. On the other hand, patients with neurological presentation were less likely to have an enlarged spleen (16.6%).

Jaundice was the most common clinical presentation in G1 (68.6%), being much less common in G3 patients (22.2%), who were more likely to present with ascites (44.4%) and lower limb oedema (55.6%). None of the latter group had hepatic encephalopathy while it occurred at presentation in 2/35(5.71%) of purely hepatic patients.

Routine liver function tests revealed a significant difference between different groups. While ALT was highest in asymptomatic patients, AST was highest in G1 and G3 patients and lowest in purely neurological cases. Serum bilirubin and albumin were most deranged in G1 patients. INR was highest in patients with combined presentation (table 3).

Liver biopsy was performed in 39 patients, identifying cirrhosis histologically in all biopsies from G3 (4/4) and nearly two thirds of the G1 patients (7/11). Liver biopsy was also performed in 20 asymptomatic children of whom 45% revealed cirrhosis. Half of biopsied patients with purely neurological disease (2/4) had either chronic hepatitis or cirrhosis (25% each) (table 3). Thirteen children with cirrhosis were ≤ 10 years old (data not shown).

Of all the hepatic patients (G1 and G3), 13/44 (29.5%) presented with ALF. Male gender predominated in patients with ALF. Median age of presentation of the ALF group (9 ± 3 years) was lower than that of AH (11 ± 4 years) and CLD (10.5 ± 4 years) (statistically not significant). KF ring was present in all tested acute patients, whether compensated or not. It was also positive in 19 out of 20 patients with CLD (table 4) and four of the asymptomatic patients (table 3).

**Table 4:** Comparison between different types with hepatic involvement (G1 and G3)

	Chronic LD	Acute hepatitis	ALF	*P *value
				
*No. (%)*	25/44 (56.8)	6/44 (13.6)	13/44 (29.5)	
***Male/Female***	13/12	3/3	11/2	0.178

***Age range (yrs)***	6-15	9-15	6-13	
***Mean age (yrs)± SD***	10.7 ± 2.8	11.57 ± 2.15	9.33 ± 2.01	1.49
***Median age (yrs) *± *IR***	10.5 ± 4	11 ± 4	9 ± 3	

***Positive KF ring No. (%)***	19/20 (95%)	6/6 (100)	10/10 (100)	0.276

SD: standard deviation, IR: interquartial range, KF: Kayser-Fleisher

The most common neurological manifestations were tremors and dysarthria. MRI brain revealed variable involvement of basal ganglia and cerebellum.

Renal manifestations were noted in 10 patients (renal stones in 6 patients and nephropathy in 4). Recurrent urinary tract infections (UTI) were present in 4 patients. Other extra hepatic manifestations detected in 13 patients were: recurrent abortion in 2 females, gallstones in 5 patients and arthralgia in 6. Co-morbidities present in 6 patients included hepatitis A, B, C in three patients (each had one infection) and bilharziasis in three patients.

Sequencing of *ATP7B *gene was performed as previously described in Abdelghaffar et al. 2008 in 64 patients to genetically characterize this cohort [9]. Molecular genetic characteristics are illustrated in (table 5).

**Table 5:** Homozygous mutations detected in children with Wilson disease in this study

Mutation	Type of mutation	No. of patients	No. of chromosomes	Form of WD
c.507delA, p.Gly170fs*	deletion/frameshift	1	2	H (CLD)
c.1186G > T, p.Glu396X*	Nonsense	3	6	2 C (CLD)
				N
c.330delA, p.Gln110fs*	deletion/frameshift	2	4	C (CLD)
c.1646T > C, p.Leu549Pro*	Missense	1	2	C (CLD)
c.1707+5G > A, IVS4+5G > A*^1^	Splice	2	4	2 C (CLD)
**c.1924G > C, p.Asp642His**	Missense	1	2	H (CLD)
**c.2108G > A, p.Cys703Tyr**	Missense	2	4	H (CLD)
				H (ALF)
**c.2304dupC, p.Met769fs**	insertion/frameshift	1	2	A
**c.2293G > A, p.Asp765Asn**	Missense	1	2	H (CLD)
**c.2532delA**, **p.Val845fs**	deletion/frameshift	2	4	H (CLD)
c.2993G > A, p.Gly998Asp*	Missense	1	2	Combined
**c.2930C > T, p.Thr977Met**^1^	Missense	2	4	H (CLD)
**c.2997dupC, p.Gly1000fs**	insertion/frameshift	1	2	H (ALF)
**c.3207C > A, p.His1069Glu**	Missense	3	6	H (CLD)
c.3373_3377delAGTCAinsTCT, p.His1126fs*	deletion-insertion/frameshift	4	8	H (CLD)
**c.3620A > G, p.His1207Arg**^1^	Missense	1	2	A
**c.3904-2A > G, IVS18-2A > G**	Splice	4	8	H (ALF)
				C (CLD)
				H (CLD)
				C (CLD)
**c.3809A > G, p.Asn1270Ser**	Missense	2	4	C (CLD
**c.3818C > T, p.Pro1273Leu**	Missense	3	6	H (CLD)
c.3731delT, p.Leu1244fs*	deletion/frameshift	1	2	H (ALF)
c.3994A > G, p.Asn1332Asp*	Missense			C (CLD)
**c.3955C > T, p.Arg1319X**	Nonsense	3	6	H (ALF) H (CLD)
c.4021G > C, p.Gly1341Arg*	Missense	2	4	H (CLD)
**c.4022G > A, p.Gly1341Asp**	Missense	1	2	H (CLD)
**c.4301C > T, p.Thr1434Met**	Missense	1	2	C (CLD)
c.4230G > A, p.Trp1410X*	Nonsense	1	2	H (CLD)
**c.3734G > T, p.1245L**	Missense	1	2	C (CLD)
**c.3188C > T, p.Ala1063Val**	Missense	1	2	N
				H (CLD)
**c.2049_2053delCCTGGinsTTTC, p.Val683_Leu684delinsVal**	deletion-insertion/frameshift	1	2	H (ALF)
**c.2332C > G, p.Arg778Gly**	Missense	2	4	H (CLD)
**c.2450delA, p.Glu817fs**	deletion/frameshift	1	2	C (CLD)
**IVS20+6T > C**	Splice	1	2	C (CLD)
**c.2231T > C, p.S744P**	Missense	1	2	H (CLD)

^1^Suspected disease causing variants

(*): novel mutations detected in this study

*genbank sequences (NM_000053.2)

A: asymptomatic, H: hepatic, N, neurological, C: combined, AH: acute hepatitis, CLD: chronic liver disease. ALF: acute liver failure

### Treatment and follow up

62 of the patients were followed up for a mean period of 58.9 ± 6.4 months (range: 14 days -17 years).

***Overall, side effects ***of D-PCA were encountered in 16 out of the 52 patients on long-term chelation (30.76%) (6 patients from G1, 3 from G2, 5 from G3 and 2 from G4). Hematuria was the commonest side effect occurring in 9 patients. It was accompanied by albuminuria in 3 patients. Deterioration of neurological symptoms occurred in 3 patients (one of whom was pregnant). Two patients developed leucopenia, while an allergic skin reaction, thrombocytopenia, loss of hair and itching occurred in one patient each.

D-PCA had to be completely discontinued in only 6 patients (11.5%). Four of them (three from the same family) developed progressive hematuria and albuminuria while in the other two, marked deterioration of neurological symptoms occurred. The rest of the side effects reverted by decreasing the dose of D-PCA.

Zinc sulfate as monotherapy was used in 9 patients (in 3 asymptomatic and in the 6 for whom D-PCA had to be discontinued). The only side effects noted were vomiting and epigastric pain, which improved when zinc was taken with little protein.

Six patients successfully conceived (5 from G4 and 1 from G3). Treatment was switched during pregnancy to zinc sulfate. Four had normal babies. One developed neurological manifestations during pregnancy. She was non-compliant on zinc and delivered a girl in whom Budd-Chiari syndrome was diagnosed at the age of 6 months. One patient delivered a baby with Fallot's tetralogy. She was already on zinc sulfate as a sole treatment when she got pregnant but she was scarcely adherent to therapy. Another stopped treatment after delivery and this was followed by recurrent abortions.

### Follow up and compliance

**Figure 1:**
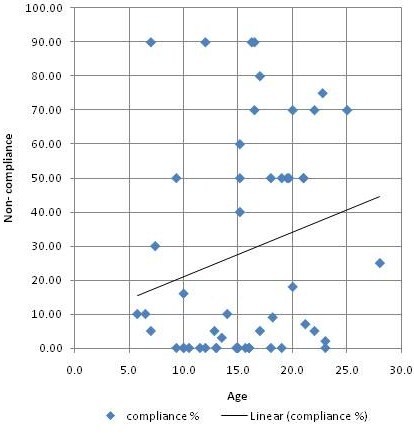
**Scatter chart showing that non compliance increased with increasing age**.

**Figure 2:**
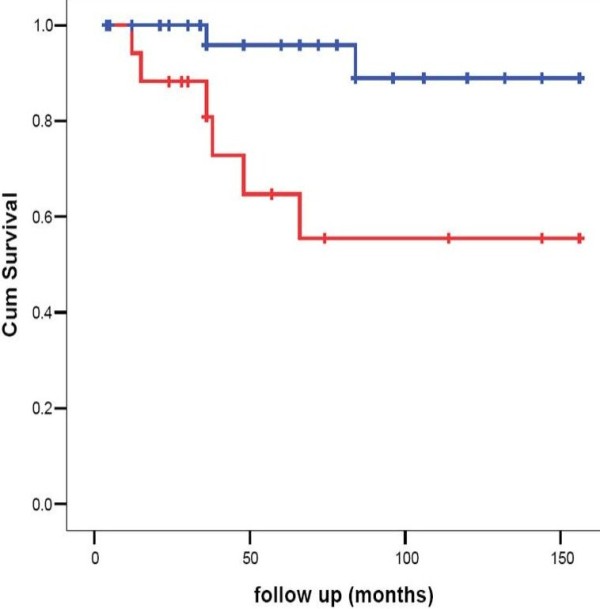
**Kaplan Meier curve showing a significant effect of compliance on survival (Blue line: non-compliance < 50% of follow up time**. Red line: non-compliance ≥ 50% of follow up time).

76.1% of the patients were compliant to therapy; compliance did not significantly differ in relation to the type of presentation (data not shown). Non-compliance increased with increasing age (figure (1). Kaplan Meier curve showed a significant effect of compliance on survival (figure 2).

### Treatment outcome

One patient out of the 62 was lost to follow up and 6 patients (5 males and 1 female) died within two weeks of presentation due to fulminant hepatitis. Forty-one out of the remaining 55 patients (74.5%) had a stable or improved course while four patients got worse; two of them were asymptomatic at diagnosis. Worsening was in the form of developing neurological symptoms or deterioration of liver disease, all four were non-compliant to therapy. Late mortality occurred in 10 patients (6 males, 3 females) (18.2%).

#### Treatment outcome of asymptomatic patients

Of the 22 asymptomatic patients who were followed up, 19 had either a stable (5) or an improved course as regards liver enzymes (14) while 2 patients got worse due to non-compliance (one developed severe neurological manifestations, and the other had deterioration of liver disease). Outcome in relation to different groups is presented in table (6).

**Table 6:** Outcome of the studied patients

	G1 (27)	G2 (4)	G3 (8)	G4 (22)	Total (61)
***Stationary***	2	1	3	5	11 (18%)

***improved***	11	2	3	14	30 (49.2%)

***worse***	2	0	0	2	4 (6.5%)
***Early death***	6	0	0	0	6 (9.8%)

***Late death***	6	1	2	1	10 (16.4%)

### Overall mortality

**Figure 3:**
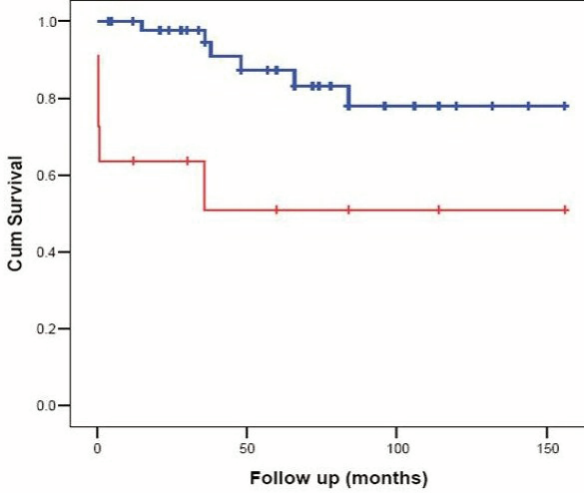
**Kaplan Meier curve showing a significant effect of Dhawan et al **[8]**prognostic score on survival (red line: prognostic score ≥11**. blue line: prognostic score <11)

Sixteen patients died in the course of disease. Early death due to ALF occurred in 6 patients and late death in 10 patients (decompensated cirrhosis in 7, bleeding esophageal varices in one patient and sepsis in two patients). Using the prognostic score proposed by Dhawan et al., 2005 [8], all patients with early death had a score of ≥ 11 while only one patient with late death had a prognostic score of ≥ 11 at presentation, figure (3). Yet, 2 out of 8 patients who had a prognostic score > 11 at presentation responded well to chelation therapy. Late death was significantly related to compliance.

## Mutational analysis

Sequencing of the *ATP7B *gene was performed in 65 patients. Mutations within the gene were identified in 64 of them and in only one patient no mutations were detected (he was excluded from the study). Eleven mutations were novel (table 5). Fifty four patients (85.7%) had homozygous mutations while only 9 (14.3%) were compound heterozygous. For better genotype-phenotype analysis, only homozygous mutations were considered for further analysis:

- 25 patients (46.3%) had missense, 14 had frameshift (25.9%), 8 had nonsense (14.8%) and 7 had splice site mutations (12.9%).

- Mean age of onset in patients with protein-truncating mutations was 9.9 years while that in patients with missense mutations was 10.46 years.

- 37.9% (11/29) of patients with protein-truncating mutations were ≤ 8 years compared to 32% (8/25) of patients with missense mutations.

- Death occurred in 17.2% (5/29) of patients with protein-truncating mutations compared to only 8% (2/25) of patients with missense mutations. Patients with protein-truncating mutations mostly died from ALF (4/5).

- Six out of 10 homozygous patients with ALF had a mutation resulting in a truncated protein (frameshift (40%), nonsense (10%) or splice site mutation (10%). Death occurred in 4 out of these 6 (66.6%) compared to only one out of the other 4 with missense mutations (25%)).

- 70% of patients who presented or eventually developed neurological symptoms had a protein-truncating mutation (25% splice site, 20% frameshift, 25% nonsense).

- Splice site mutations were present in 7 patients, 5 of them eventually developed neurological symptoms

- The most common mutation found was c.3904-2A > G, IVS18-2A > G, which was found in 5 patients, 3 of them were from the same family. This was followed by c.3373_3377delAGTCAinsTCT, p.His1126fs, which was found in 4 patients from the same family and c.3207C > A, p.His1069Glu, which was also found in 4 patients, 3 of them from the same family.

## Discussion

In this cohort of children with WD who are mostly Egyptians, there are a number of diagnostic and clinical features, which differ from those previously reported in the Western literature. The KF ring, which in absence of chronic cholestasis is the best diagnostic sign of WD, has been found in 96.4% of our children with purely hepatic manifestations. All patients with acute hepatitis as well as all patients with ALF had a KF ring. Furthermore, it was found in 17.4% of asymptomatic siblings. These figures are substantially higher than those reported in children with hepatic WD. Merle et al., 2007 reported the presence of a KF ring in 66.3% of their pediatric cohort. In most other studies, a KF ring was present in less than 50% of the patients [3,8,13]. The youngest child in this cohort positive for KF ring was only 6 years old. Although this high frequency of KF ring could indicate early onset of neurological disease, the possibility of environmental exposure to excessive amounts of copper could not be ruled out.

Only 6.5% of our patients had normal ceruloplasmin. Dhawan et al., 2005 found a normal ceruloplasmin in 20% of their pediatric cohort [8]. It seems thus that within this cohort, it is very uncommon to find symptomatic patients negative for KF ring and having a normal ceruloplasmin level, suggesting that the value given to different items in the Leipzig score might have to be modified for different ethnic groups.

Coomb's negative hemolytic anemia occurred at presentation in only 5.4% of patients, a percentage that is much less than that reported by Walshe 1987 (11%) [14], and slightly more than in Japanese patients (1.2%) [4].

Although in this study urinary copper excretion before and after D-PCA challenge yielded results diagnostic of WD in 84.2% of patients, it was performed in only 25 patients in this cohort, thus revealing the difficulty in obtaining accurate 24 hour urine collection for performing this test in children and confirming previous reports [8].

Other features characteristic of this pediatric cohort was the low age of onset of the disease. Median age of onset in our children was 10 years. Previous studies reported an onset of 12.2 years in Japan, 11 ± 7 years in Iran and 7.2 years in India [15-17]. This finding may suggest that Egyptians may have an early age of onset of WD. Whether this finding is related to the effect of environmental factors or to polymorphisms in the MURR1 gene needs further studies.

While cirrhosis is frequently found in most patients with WD by the second decade of life [18], in our study almost two thirds of cirrhotic patients were ≤ 10 years old (13/21). In addition, 45% of asymptomatic children were already cirrhotic at the time of diagnosis (as confirmed by liver biopsy) with their median age being only 8 years. The youngest patient with cirrhosis in this cohort was only 7 years old. Ascites was present at presentation in more than half of the patients with liver symptoms. Again, a figure higher than that reported in other studies [4,19]. This implies that the hepatic disease runs a more aggressive course in the Egyptian children a finding that might be related to genetic factors, undetermined environmental factors or gene modifiers.

Co-existence of WD and viral infections may lead to early onset, severe presentation and rapidly progressive liver disease [20]. True co-morbidity of WD at the time of diagnosis is rarely reported, no more frequently than expected by chance [21]. It is to be noted that in this study the three patients who had schistomiasis ran a detrimental course (one died, one developed neurological symptoms and the last had deterioration of his liver functions). To the best of our knowledge, the effect of schistomiasis on the course of WD has not been reported before.

Due to the high incidence of consanguineous marriage (78.9%) and the extended family size in our community, asymptomatic siblings constituted 35% of this cohort, thus allowing for studying the disease in its presymptomatic phase. Most of those children (81.5%) had hepatomegaly, one third had splenomegaly (37.5%) and the only disturbed LFTs were ALT/AST. In spite of being asymptomatic, yet histologically evident cirrhosis was present in 45% and a KF ring was detected in 17.4%. The youngest affected sibling was only one year old. This emphasizes the need for screening of all sibs of affected children even at a younger age than previously recommended.

Nearly one third of the patients (29.5%) with hepatic manifestations presented with ALF. A slightly higher percentage was reported by Dhawan et al. (33%) [8], yet, lower figures were reported in other studies [22]. Again, the age of onset of patients with ALF in this cohort (median: 9 years) is less than generally reported. In our cohort as well as that of Dhawn et al., there was a male predominance. This is in contrast to previous reports where females were more commonly represented among WD patients with ALF [21]. It has been suggested that the female predominance was due to the effect of sex hormone as shown in the animal model of WD [23]. Dhawan et al. attributed these contrasting results to the fact that most of their patients were prepubertal, which holds true for our group of patients as well [8].

In this study, as in others [8,24-27], D-PCA has been shown to be an effective copper chelator in both symptomatic and asymptomatic WD patients who are compliant to treatment.. Side effects were observed in 30.76% and were severe enough to discontinue the drug in 11.5%. Such figures are slightly less than those reported by others for both adults [3] and children [8,28]. In Egypt, the only available decoppering agent is D-PCA, so we tried hard to limit its side effects by starting therapy with a very small dose that was very gradually increased to reach the maximum desired dose meanwhile giving zinc sulphate starting with a maximal dose and gradually reducing it as D-PCA was increased.

Of the children born to WD mothers one suffered from Fallot's tetralogy and another was diagnosed as Budd-Chiari syndrome at 6 months of age. In both cases, the mother did not receive D-PCA during pregnancy and they both were totally non-compliant on zinc therapy. This may implicate intrauterine exposure to excess copper, a teratogenic substance, as the cause in both children.

Zinc monotherapy has been shown to be an effective non-toxic alternative to D-PCA in asymptomatic siblings [29]. It was approved in 1997 by the US FDA as maintenance therapy. Yet some reports indicate its efficacy also as first line treatment of symptomatic patients [7]. Three of the asymptomatic children in this study have been effectively treated with zinc monotherapy. It was also used in the six patients for whom D-PCA had to be discontinued and in all it was effective. The mechanism of action of Zinc is different from that of D-PCA as it inhibits copper absorption by inducing enterocyte metallothionin. It may also induce hepatocyte metallothionin [30] and can generate a negative balance for copper thereby removing stored copper [31]. We used the combination of Zinc sulphate (a single midday dose) and D-PCA for our patients, spacing them at least 6 hours apart. Although the use of a single midday zinc sulfate dose decreases the chance of non compliance, its effectiveness in the production of a negative copper balance is uncertain.

Six patients in this series died within two weeks of presentation. They all had a score >11 in the new Wilson predictive index proposed by Dhawan et al. 2005. None of the patients with score <11 experienced early death, yet two patients with a score >11 survived on medical management without transplantation. In their original description of the score, Dhawan et al. 2005 indicated that a score >11 was predictive for death with a sensitivity of 93%, specificity of 98% and positive predictive value of 88% [8].

During follow up ten patients died, in six of whom death resulted from hepatic decompensation. All six were non-adherent to therapy. The adverse effect of non- compliance to therapy on survival has been clearly demonstrated in the Kaplan Meier survival curve. Nearly 24% of our patients were non adherent to therapy more than 50% of the time. Brewer et al. (1994) found that about 10% of the patients had serious non adherence problems and another 20% had episodic non adherence when followed up for 5-10 years [29]. Arnon et al. (2007) reported that poor adherence to therapy might be responsible for the persistence of elevated ALT [10]. The patients in this study who developed liver decompensation could not be rescued since liver transplantation became available in Egypt only since late 2001.

Homozygous mutations were present in 54 patients (85.7%). This high percentage may be explained by the high percentage of consanguinity in our study (78.9%) compared to the consanguinity in the Egyptian population (32-35%) [32]. In spite of the high percentage of homozygous mutations, it was difficult to find genotype-phenotype correlations due to the large number of mutations detected. Heterogeneity of the Egyptian population with respect to ethnicity may justify the presence of such big number of different mutations that may also reflect a high carrier rate for WD in our population.

Protein truncating mutations profoundly affect gene functions [27,33]. Patients with protein-truncating mutations in this study had slightly earlier age of onset, died earlier from ALF and neurological symptoms were more common among them.

## Conclusions

Egyptian children with WD present with early KF rings, and early onset of liver and neurological disease. The mutation spectrum identified differs from that observed in other countries. This study offers screening strategies for WD in Egypt, which may not depend on genetic testing.

## List of abbreviation

AH: Acute hepatitis; ALF: Acute liver failure; CLD: Chronic liver disease; D-PCA: D- Penicillamine; KF: Kayser-Fleischer; LFT: Liver function tests; UTI: Urinary tract infection; WD: Wilson disease

## Competing interests

The authors declare that they have no competing interests.

## Authors' contributions

TYA: designed the study, diagnosed and followed the patients, analyzed and interpreted the data, and participated in drafting the manuscript. SME: participated in designing the study, diagnosed and followed the patients, analyzed and interpreted the data, and participated in drafting the manuscript. SE: helped in diagnosis, following up the patients, and helped in collecting the data. AS: helped in diagnosis, following up the patients, and helped in collecting the data. ES: helped in interpretation of molecular genetic data. HS: carried out the molecular genetic studies and helped in drafting the manuscript. All authors read and approved the final manuscript.

## Pre-publication history

The pre-publication history for this paper can be accessed here:

http://www.biomedcentral.com/1471-2431/11/56/prepub
